# Identification and Characterization of the miRNA Transcriptome of *Ovis aries*

**DOI:** 10.1371/journal.pone.0058905

**Published:** 2013-03-13

**Authors:** Shifang Zhang, Fuping Zhao, Caihong Wei, Xihui Sheng, Hangxing Ren, Lingyang Xu, Jian Lu, Jiasen Liu, Li Zhang, Lixin Du

**Affiliations:** 1 National Center for Molecular Genetics and Breeding of Animal, Institute of Animal Sciences, Chinese Academy of Agricultural Sciences, Beijing, China; 2 College of Animal Science and Technology, Beijing University of Agriculture, Beijing, China; 3 Chongqing Academy of Animal Sciences, Chongqing, China; French National Center for Scientific Research - Institut de biologie moléculaire et cellulaire, France

## Abstract

The discovery and identification of *Ovis aries* (sheep) miRNAs will further promote the study of miRNA functions and gene regulatory mechanisms. To explore the microRNAome (miRNAome) of sheep in depth, samples were collected that included eight developmental stages: the longissimus dorsi muscles of Texel fetuses at 70, 85, 100, 120, and 135 days, and the longissimus dorsi muscles of Ujumqin fetuses at 70, 85, 100, 120, and 135 d, and lambs at 0 (birth), 35, and 70 d. These samples covered all of the representative periods of *Ovis aries* growth and development throughout gestation (about 150 d) and 70 d after birth. Texel and Ujumqin libraries were separately subjected to Solexa deep sequencing; 35,700,772 raw reads were obtained overall. We used ACGT101-miR v4.2 to analyze the sequence data. Following meticulous comparisons with mammalian mature miRNAs, precursor hairpins (pre-miRNAs), and the latest sheep genome, we substantially extended the *Ovis aries* miRNAome. The list of pre-miRNAs was extended to 2,319, expressing 2,914 mature miRNAs. Among those, 1,879 were genome mapped to unique miRNAs, representing 2,436 genome locations, and 1,754 pre-miRNAs were mapped to chromosomes. Furthermore, the *Ovis aries* miRNAome was processed using an elaborate bioinformatic analysis that examined multiple end sequence variation in miRNAs, precursors, chromosomal localizations, species-specific expressions, and conservative properties. Taken together, this study provides the most comprehensive and accurate exploration of the sheep miRNAome, and draws conclusions about numerous characteristics of *Ovis aries* miRNAs, including miRNAs and isomiRs.

## Introduction

MicroRNAs (miRNAs) are a class of endogenously small noncoding RNAs that are about 22 nucleotides (nt) in length. Lee et al. [1] first found *lin-4*, which controls the time-ordered development of *Caenorhabditis elegans*. Then Reinhart et al. [2] found another small RNA that possessed a posttranscriptional regulatory function, which was named let-7. In the following years, more and more researchers have successfully discovered this kind of RNAs and named these small RNAs miRNAs, which are noncoding and have specific temporal and spatial expressions. MiRNAs are extensively present in various kinds of animals, plants, and viruses [3]; they mainly cause specific genes to become silent through complementary base pairing with the target gene’s 3′-untranslated regions (3′-UTR) of mRNAs. Bioinformatics predictions indicate that mammalian miRNAs could regulate about 30% of protein-coding genes [4]. Many studies have revealed that miRNAs play crucial roles in a broad range of biological processes, including development, differentiation, and tissue morphogenesis, and also in several types of diseases such as carcinogenesis and viral infections [5]. Taking full advantage of different sequencing platforms, researchers have discovered many new miRNA candidate genes [6]–[7] in fishes [8], chickens [9], mice [10], and mammals. Further study of miRNAs will prove beneficial to expanding our understanding of physiological and pathological mechanisms in organisms, and will provide a theoretical basis for the diagnosis and treatment of diseases.

Sanger miRBase v17.0 (April 2011) includes 16,772 published entries representing hairpin precursor miRNAs, expressing 19,724 mature miRNA products in 153 species. The data set includes 662 precursor miRNAs from cattle, but only 55 precursor miRNAs from sheep. The sheep genome project is currently in progress and the genome size is estimated at 2.97 Gb. The genome project will lay the foundation for miRNA mapping and miRNA function assessments through molecular regulatory networks.

Texel sheep are typically “double-muscled” sheep, while Ujumqin sheep are the native fleshy-fat sheep. The two sheep breeds represent phenotypic extremes and are therefore appropriate animal models for the research of *Ovis aries* growth and development. The maximum myofiber complement of a sheep fetus is achieved during the second half of gestation; specifically, days 70, 80, 100, 120 and 130 are the most important [11]. Our laboratory has completed expression profile microarrays of fetus skeletal muscles in Texel and Ujumqin sheep. However, the mechanism by which miRNAs regulate myofiber proliferation in sheep during these periods is not clear. Hence, this study carried out additional miRNA deep sequencing to obtain miRNA expression profiles of skeletal muscles, which will contribute to the identification of differences between the mechanisms in Texel and Ujumqin muscle development.

To encompass the main morphological and physiological changes of *Ovis aries* that occur during growth and development from gestation to 2 months after birth, longissimus dorsi muscles of Texel and Ujumqin sheep were obtained from eight representative developmental stages. Texel and Ujumqin libraries were individually sequenced using a GAIIx instrument. After analysis, the two miRNA libraries generated 35,700,772 reads that corresponded to 2,048,650 high-quality reads.

## Materials and Methods

### Ethics Statement

Texel and Ujumqin sheep were obtained from a sheep stud farm located in Youyu, Shan Xi Province. All experimental and surgical procedures were approved by the Biological Studies Animal Care and Use Committee, Shanxi Province, Peoples Republic of China. The ewes were housed in one group and were fed according to the nutrient requirements of sheep established by the National Research Council in 1985; the feeding was in line with the Instructive Notions with Respect to Caring for Laboratory Animals that was published in 2006 by the Science and Technology Department of China (Approval No. S20072911).

### Sample Collection and RNA Extraction

Five Texel and eight Ujumqin ewes of similar age (3–5 years old), body weight (50–55 kg), and body size were selected. The estrus of the 13 ewes was synchronized and artificial insemination was completed. The date of artificial fertilization was used as day zero of gestation. Texel and Ujumqin fetuses were selected from five stages in utero by surgery: 70, 85, 100, 120, and 135 days after mating, abbreviated below as 70 d, 85 d, 100 d, 120 d, and 135 d, respectively. Ujumqin lambs were euthanized at three stages after birth: 0, 35, and 70 days, abbreviated below as 0 d, 35 d, and 70 d, respectively. The longissimus dorsi muscles (LM) of these 13 animals were dissected and quickly stored in liquid nitrogen.

Total RNA was separately isolated and purified from 13 frozen LM samples using a special Animal Tissue RNA Purification Kit (Product #TRK-1002; Qiagen, Hilden, Germany). After purification, RNA purity was assessed using a spectrometer (Nanodrop, Wilmington, DE, USA) at ratios of OD_260_/OD_280_ above 1.8. This showed that RNA was high purity. And RNA integrity assessing was conducted using agarose gel electrophoresis (AGE). Two 28S/18S rRNA bands were seen clearly in AGE without other bands. The results illustrated that RNA was high integrity and could be further used for Solexa deep sequencing.

### Library Construction and Deep Sequencing

For Texel sample set, equal quantities of total RNA isolated from the longissimus dorsi muscles of Texel fetuses at 70, 85, 100, 120, and 135 d were pooled (see Table S1). For Ujumqin sample set, equal quantities of total RNA isolated from the longissimus dorsi muscles of Ujumqin fetuses at 70, 85, 100, 120, and 135 d, and lambs at 0 (birth), 35, and 70 d were pooled. Two small RNA libraries were generated from equal quantities of total RNA representing Texel and Ujumqin sample sets using Illumina Truseq Small RNA Preparation kits. The two purified cDNA libraries were used for cluster generation with Illumina’s Cluster Station and then sequenced on an Illumina GAIIx instrument. Raw sequencing reads were obtained using Illumina’s Sequencing Control Studio software version 2.8 (SCS v2.8) following real-time sequencing image analysis and base-calling using Illumina’s Real-Time Analysis version 1.8.70 (RTA v1.8.70). The extracted sequencing reads were used in data analysis.

### Data Analysis

We used a proprietary pipeline script, ACGT101-miR v4.2, to analyze the sequencing data. The data analyses were performed as follows:

1. Obtaining Mappable Sequences from Raw Sequencing Data. “Impure” sequences due to sample preparation, sequencing chemistry and processes, and the optical digital resolution of the sequencer detector were removed using a series of digital filters. The remaining sequencing sequences (sequ seqs, between 15 and 32 nt in length) were grouped into families (unique sequences).
2. Mapping miRNA-mappable Unique Sequences (Unique Seqs) to Pre-miRNAs and the Genome. The number of selected mammalian pre-miRNAs in the latest version of miRBase (v17.0) was 6,752, among which the number of *Ovis aries* pre-miRNAs was 55. Unique seqs were aligned against pre-miRNAs of Mammalia.
3. Mapping Unique Sequences to Selected Databases.

For conciseness, the classification of six generated sequence groups was summarized in Table 1.

**Table 1:** Classification of six generated sequence groups by data analysis.

Classification	Group		Sequence
Mapped to selectedpre-miRNAs in miRbase v17.0	Group 1	Group 1a	Mapped to *Ovis aries* known pre-miRNAs
		Group 1b	Mapped to Mammalia known pre-miRNAs which could be mapped to *Ovis aries* genome
	Group 2	Group 2a	Mapped to known pre-miRNAs of Mammalia and *Ovis aries* genome; within hairpins
		Group 2b	Mapped to known pre-miRNAs of Mammalia and *Ovis aries* genome; no hairpins
	Group 3	Group 3a	Mapped to known pre-miRNAs and miRNAs of Mammalia but unmapped to *Ovis aries* genome
		Group 3b	Mapped to known pre-miRNAs of Mammalia but unmapped to *Ovis aries* genome
Unmapped to selectedpre-miRNAs in miRbase v17.0	Group 4	Group 4a	Unmapped to known miRNAs but mapped to *Ovis aries* genome and within hairpins
		Group 4b	Unmapped to known miRNAs but mapped to *Ovis aries* genome and without hairpins
Mapped to other defineddatabases	Group 5	Others	Mapped to other defined databases, such as mRNA, RFam, or Repbase
	Group 6	Nohit	Unmapped to Groups 1, 2, 3, 4, or any of the defined databases

### Prediction of *Ovis aries* miRNA Target Genes

In principle of miRNA target with mRNA, we downloaded 3536 *Ovis aries* mRNA sequences from NCBI database. Then we extracted 2258 conserved 3′UTR sequences of *Ovis aries* genes. Applied target prediction tool which was TargetScan database (http://www.targetscan.org/) to predict the potential *Ovis aries* target genes of 89 differentially expressed miRNAs between Texel and Ujumqin sheep (see Table S2). A series of rules were used: (1) a perfect Watson-Crick match between target gene and miRNA at 2–8 positions (numbered from the 5′ end); (2) one G:U pair in the seed match was allowable; (3) the threshold for the minimum context score percentile of the seed match was 50.

### Quantitative Real-time PCR of *Ovis aries* miRNAs

The pool RNA used in deep sequencing was reverse transcribed by stem-loop antisense primers (see Table S3), respectively. The expression of 25 selected miRNAs was determined using the Quantitative real-time PCR conducted on the ABI PRISM® 7900HT Real-time PCR Detection System, with 5S ribosomal RNA as the internal control gene. For cDNA synthesis, 250 ng total RNA and 0.5 µL RT primer (2 µmol/L) were mixed and ddH_2_O was added to adjust the total volume to 3.0 µL, the mixture was incubated at 65°C for 10 min and snapped on ice for 3 min. Then a 5.0 µL reaction mixture contained 3.0 µL denatured total RNA and RT primer (2 µmol/L), 0.25 µL dNTP (10 mmol/L each), 1.0 µL 5×RT buffer, 0.25 µL RNase inhibitor (40 U/µL) and 0.5 µL M-MLV (200 U/µL) and reaction conditions were 42°C for 60 min, and 70°C for 15 min and hold at 4°C. The quantitative real-time PCR (qRT-PCR) reaction mixture (20 µL) contained 0.5 µL RT product, 0.8 µL primer mix (10 µmol/L), 10.0 µL 2 X SYBR Green Mix With ROX, 8.7 µL ddH_2_O and cycling conditions were 50°C for 2 min; 95°C for 2 min; and followed by 39 cycles of 95°C for 15 s, and 60°C for 30 s. Melting curves were analyzed after amplification. All reactions were run in triplicate. We analyzed relative quantification results by the 2 ^−ΔΔ^CT method, and statistically significant differences between Texel and Ujumqin were examined using a t-test.

## Results

### Solexa Sequencing Data

Raw sequencing reads were obtained using Illumina’s Sequencing Analysis software; 35,700,772 raw reads were generated in the two libraries. We used ACGT101-miR v4.2 to analyze the sequencing data (see Figure S1). By removing various un-mappable sequencing reads from the raw sequence reads, or the sequ seqs, the remaining sequ seqs (filtered sequ seqs with length between 15 and 32 nt) were further classified as mappable sequences (see Figure S2). After collating and mapping sequences to the constructed databases (sheep genome project and Mammalia miRBase), overlapping reads were gathered into clusters. Reads within each cluster were sorted by the number of mismatched bases, and counts were blasted to the genome. The same mismatched reads were arranged in line with the expression of the reads.

The 2,319 unique miRNAs were divided into three groups: high count miRNAs (counts ≥4,718), middle count miRNAs (10≤ counts <4,718), and low count miRNAs (0≤ counts <10). Each library included 2,319 miRNAs that represented 10,940,530 total average counts (see Table S4). High count miRNAs accounted for 97.99% of the total miRNA expression on average, but only for 4.87% of the types of miRNA, suggesting that a few miRNAs played a leading role as candidate miRNAs. The average counts for high count miRNAs reached 94,878.89, whereas the average counts for low count miRNAs were only 1.38, indicating that only a few types of miRNA comprised the majority of sequences.

In our data, RFams (RFam: rRNA, tRNA, snRNA, snoRNA, and others) represented the maximum proportion of the known classes of RNA sequences (3.23%) and accounted for 5.99% of the total variation. However, mRNA represented only 0.12% of the total number of sequences and only 2.05% of the total variation (see Figure S3 and Table S5). The proportion of long-chain RNAs was very low, demonstrating that our constructed RNA libraries were of high quality and that the reliability of the sequence results was high. Nohit group reads were un-mapped to selected pre-miRNAs (in miRBase v17.0), mRNA, Rfam, Repbase, or the sheep genome. Because the sheep genome was not complete to date, 30.27% reads were classified as Nohit group reads. The sheep genome project is currently in progress and will lay the foundation for miRNA mapping from Nohit group reads.

### Mappable Sequences

**Figure 1:**
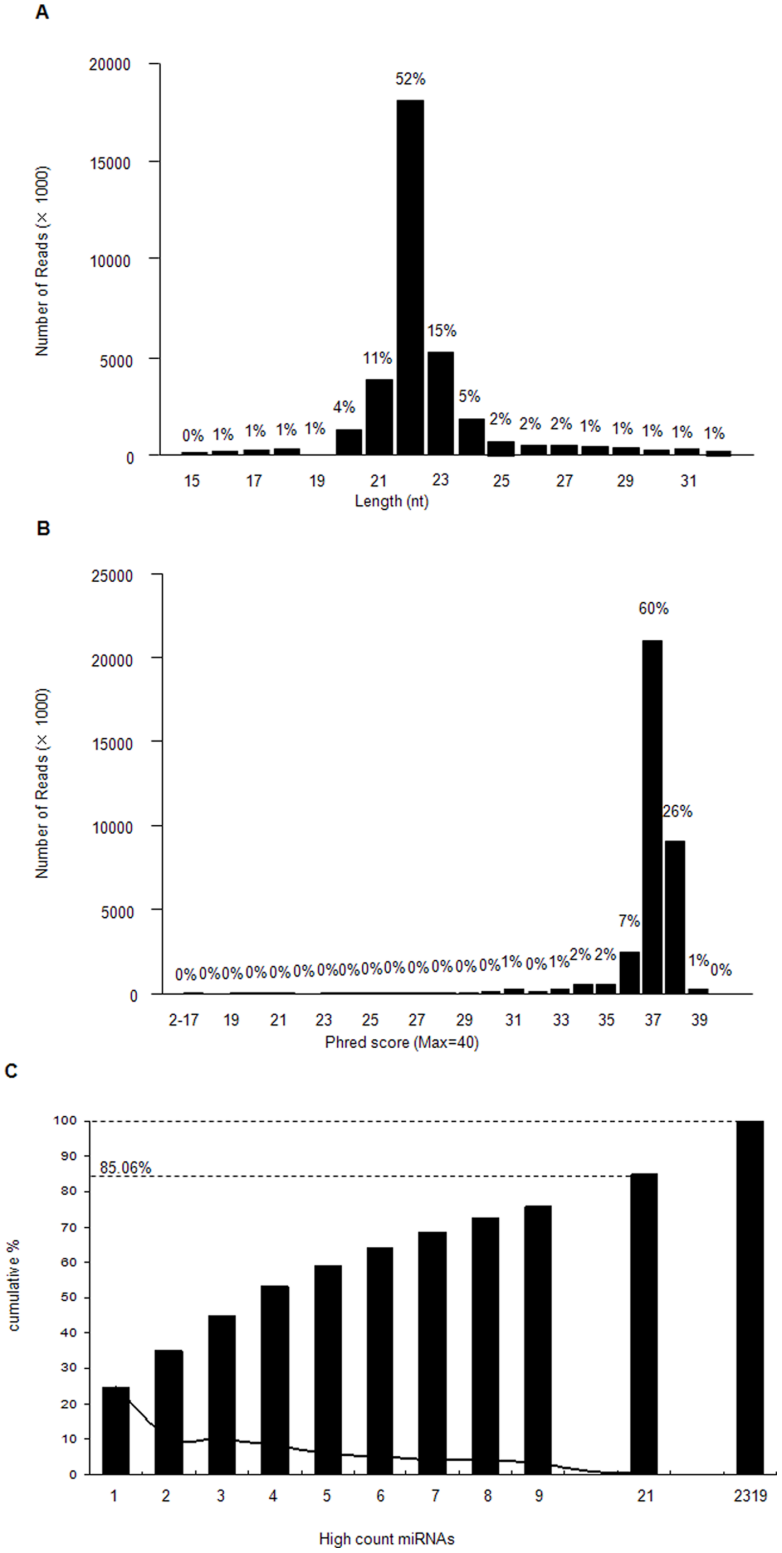
The length and count distribution of the sequenced sequences. A. Length distribution of reads after 3ADT (3′adapter) cut. B. Histogram of the average phred score^1^ per base in a read after 3ADT cut. ^1^ Phred score larger than 30 stands for probability of incorrect base calls less than 1 in 1,000 (above 99.9% accuracy) in one sequencing read. C. Histogram of the top 21 high count miRNAs (X-axis) versus their cumulative % (Y-axis) in total average counts of the two small RNA libraries. The cumulative % of the top 21 high count miRNAs and the overall 2,319 unique miRNAs were shown as the dashed horizontal line at 85.06% and the dashed horizontal line at 100%, respectively, and the % of the individual miRNA was expressed as the black line.

After excluding all un-mappable sequencing reads, the remaining sequ seqs were termed mappable sequences. The analysis of mappable sequences illustrated that the length distribution peaked at 21 and 23 nt, and 22 nt sequences accounted for the maximum percentage (51.69%). These results are typical products of Dicer incisions (see Figure 1A) [12]. Similar variation in miRNA length has been repeatedly detected in deep sequencing results of other species. The average phred score per base in a read after a 3′ adapter (3ADT) cut at base 37 accounted for the largest proportion of sequences (60%) (see Figure 1B). Both of these results confirmed that the sequ seqs were of high quality.

### Classification of *Ovis aries* miRNAs

Variation in the mappable sequences was concentrated at both the 3′ and 5′ ends, which produced multiple mature variants that were named isomiRs, as previously described [13]. Measurements of the abundance of a miRNA/miRNA* using the sum of the total isomiR sequence counts correlated well with the expression level of the most abundant miRNA/miRNA* sequence [14]. Thus, in our study, we focused on the most abundant miRNA/miRNA* sequence for the differential abundance analysis.

First, sequences were mapped to the constructed databases (see Figure S1). Then, sequences were sorted by their mapping to known ovine miRNAs and other known mammalian miRNAs, and their locations within the ovine genome. For conciseness, the seven types of obtained *Ovis aries* miRNAs were summarized in Table 2.

**Table 2:** Summary of the seven types of obtained *Ovis aries* miRNAs.

Classification	Type	miRNA	#miRNAs detected	#Clusters	#Known_pre-miRNAs	#Known_miRNAs	SupplementaryTable
Known miRNAs	Group 1a	of *Ovis aries*	96	49	276	377	Tables S6, S7
	Group 1b	of Mammalia, but novel to *Ovis aries*	439	275	1685	2122	Tables S8, S9
Predicted miRNAs	Group 2a	Mapped to known pre-miRNAs of Mammalia and *Ovis aries* genome; within hairpins	32	32	51	72	Tables S10, S11
	Group 2b	Mapped to known pre-miRNAs of Mammalia and *Ovis aries* genome; no hairpins	272	249	161	182	Tables S12, S13
	Group 3a	Mapped to known pre-miRNAs and miRNAs of Mammaliabut unmapped to *Ovis aries* genome	414	318	1351	1563	Tables S14, S15
	Group 3b	Mapped to known pre-miRNAs of Mammalia but unmappedto *Ovis aries* genome	89	77	638	682	Tables S16, S17
	Group 4a	Unmapped to known miRNAs but mapped to *Ovis aries* genome and within hairpins	1685	1470	0	0	Tables S18, S19

Note: #miRNAs detected: the number of detected miRNAs; #Clusters: the number of clustered sets of these detected miRNAs; #Known_pre-miRNAs: the number of mapped pre-miRNAs in miRbase v17.0; #Known_miRNAs: the number of known miRNAs coded by these mapped pre-miRNAs.

In total, 2,319 pre-miRNAs, expressing 2,914 miRNAs, were detected in this study. Some mature miRNAs might have been from different pre-miRNAs or genome locations, and therefore, these miRNAs were suffixed behind the name (see Table S20) according to the naming rule in miRBase. The analysis of the seven types of sequenced miRNAs was summarized in Tables S5. Ninety-six known *Ovis aries* miRNAs accounted for 11.67% of the total counts; 1,685 miRNAs were mapped to the sheep genome but were not homologous with other mammals. We thus identified them as *Ovis aries-*specific miRNAs; these miRNAs only accounted for 0.43% within the 11.67% of the total counts. The remaining 1,246 miRNAs could be mapped to other mammalian pre-miRNAs, and thus were described as mammalian conserved miRNAs; the portion of these miRNAs in the total counts exceeded 52.35%. These results also indicated that miRNAs were highly conserved in this species.

Analyses of sequence results revealed that the total counts were dominated by a few miRNAs (see Figure 1C); e.g., the first nine miRNAs accounted for 75.84% of the total average counts. In addition, the first nine miRNAs were all mammalian conserved miRNAs; two of the nine were known *Ovis aries* miRNAs, while the other seven were other mammalian conserved miRNAs.

The expression of 2319 miRNAs between Texel and Ujumqin sheep was further log_2_-transformed. After analysis, 89 *Ovis aries* miRNAs were of significant difference (| log_2_ | ≥1.5) and high level (counts ≥10) in the expression (see Table S2). These 89 miRNAs were selected for further prediction of miRNA target genes.

### Known *Ovis aries* miRNAs

**Figure 2:**
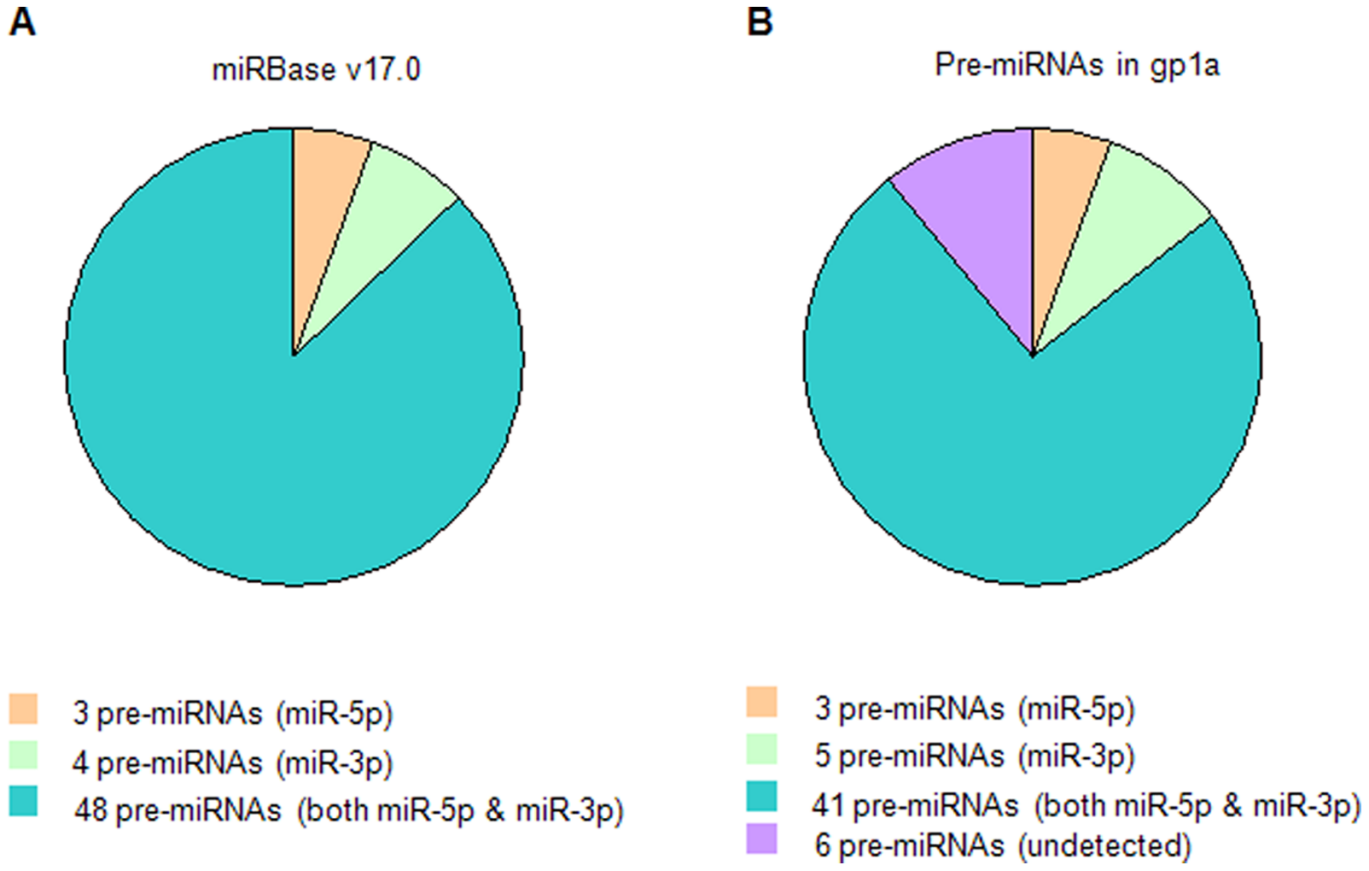
The *Ovis aries* known miRNAs. Comparison of *Ovis aries* known pre-miRNAs between miRBase v17.0 and gp1a in this study. A. The overall 55 *Ovis aries* pre-miRNAs recorded in miRBase v17.0. B. The 49 *Ovis aries* known pre-miRNAs sequenced in gp1a of this study. These pre-miRNAs in four groups: type containing miR-5p (tawny), miR-3p (light green), both miR-5p & miR-3p (aqua), and six undetected pre-miRNAs (lilac) in gp1a.

In total, 4,079,965 sequences were detected in gp1a, accounting for 11.67% of the total counts. These mappable reads were mapped to the *Ovis aries* genome and 103 *Ovis aries* miRNAs in miRBase v17.0 (see Figure 2A), and they were further classified as 96 miRNAs (see Table S6). These results indicate that miRNAs that were known and had higher expression levels were much easier to detect.

Forty-nine of 55 *Ovis aries* pre-miRNAs in miRBase v17.0 were sequenced in gp1a (see Figure 2B), and they coded 89 known miRNAs. The high coverage illustrated that the constructed RNA libraries contained the majority of known *Ovis aries* miRNAs. After further study, we discovered that 41 of 49 detected pre-miRNAs coded both the 5′ and 3′ ends of miRNAs (miR-5p and miR-3p). In many cases, miRNAs that have rapid turnover rates cannot be detected by conventional methods, although deep sequencing allows many of them to be identified.

The expression patterns of the 103 known *Ovis aries* miRNAs differed considerably in this study, ranging from a few to millions of copies. Oar-miR-127-3p and oar-miR-379-5p were the first two miRNAs and they accounted for 5.13% and 2.60% of the total mappable reads, respectively. Six new sheep miRNAs were discovered in gp1a: oar-miR-127-5p, oar-miR-136-3p, oar-miR-323b-5p, oar-miR-323c-5p, oar-miR-431-3p, and oar-miR-432-3p. They showed complementary base pairing with known *Ovis aries* miRNAs (see Table S6).

### isomiRs

The mappable reads in each group are listed based on the number of mismatched bases with known *Ovis aries* miRNAs, and reads of the same mismatched base number are also listed by the number of copies. For example, 6,849 isomiRs were detected for both the 5′ and 3′ ends of 49 pre-miRNAs in gp1a; within those, oar-mir-379 had 388 variants at the 5′ end and 96 variants at the 3′-end, respectively (see Table S7). These results fully illustrate that known miRNAs were mainly present as isomiRs. Moreover, 45 isomiRs that had the maximum counts in 96 miRNA clusters were consistent with known *Ovis aries* miRNAs, and another 51 isomiRs differed by very few bases from known sheep miRNAs.

The number of isomiRs of individual miRNAs ranged from one to hundreds; e.g., oar-mir-127 had the highest diversity of isomiRs (716), and isomiRs in 24 of 49 clusters in gp1a had more than 100 variants. The variation in isomiRs was quite diverse; differences occurred at only the 5′ end, only the 3′ end or at both ends. Variation at the 5′ end might affect the seed sequence (the 2^nd^ to 7^th^ bases at the 5′ end) and could produce different miRNAs, thereby changing the target mRNA, or cause upregulation of the transcription in some regions [15].

### Candidate *Ovis aries* miRNAs

The expression levels of miRNAs discovered in this study are summarized in Table S20. We found that 94 miRNAs that corresponded to 93 unique miRNAs were known *Ovis aries* miRNAs. Another 2,820 predicted *Ovis aries* miRNAs, representing 2,226 unique miRNAs, were candidate *Ovis aries* miRNAs. Within these, 1,653 predicted candidate miRNAs, representing 1,216 unique miRNAs, could not be mapped to any known miRNAs, but they were mapped to the sheep genome and occurred within hairpins. This “PC” type miRNAs might represent *Ovis aries*-specific miRNAs. The other 1,167 miRNAs, representing 1,010 unique miRNAs, were mammalian conserved miRNAs.

The let-7 family of miRNAs comprises one of the key regulatory elements in the developmental process. The let-7 family has been studied in many species, including mammals, birds, insects, and plants. Phylogenetic analyses have demonstrated that the let-7 family is highly conserved in both sequence and function among mammals, and it plays a critical role during animal development. For example, let-7 was identified as a heterochronic switch gene. The loss of let-7 gene activity causes reiteration of larval cell fates during the adult stage, whereas increased let-7 gene dosage leads to precocious expression of adult fates during larval stages.

To date, nine kinds of let-7 family genes have been identified [i.e., let-7a, let-7b, let-7c, let-7d, let-7e, let-7f, let-7g, let-7i, and let-7j (only identified in dogs)] in mammals. Eight kinds of *Ovis aries* let-7 family genes were discovered during this research: oar-let-7a-2 (427,369.75 reads), oar-let-7a-3 (288 reads), oar-let-7b (84,793.63 reads), oar-let-7c-1 (86,997.63 reads), oar-let-7d (28,816 reads), oar-let-7e (18,198.73 reads), oar-let-7f-1 (156,359.98 reads), oar-let-7g (19 reads), and oar-let-7i (148,896.89 reads). None of these miRNAs were included in miRBase v17.0. Obvious differences were noted in the expression levels of the let-7 family. A comparison of these eight let-7 family types between *Ovis aries* and 12 other mammals illustrated that the let-7 family sequences had high similarity within Mammalia (see Figure S4). This study also found that the *Ovis aries* let-7 family miRNAs possessed the same seed sequence (5′-GAGGTA-3′).

### Chromosomal Mapping of Sheep miRNAs

**Figure 3:**
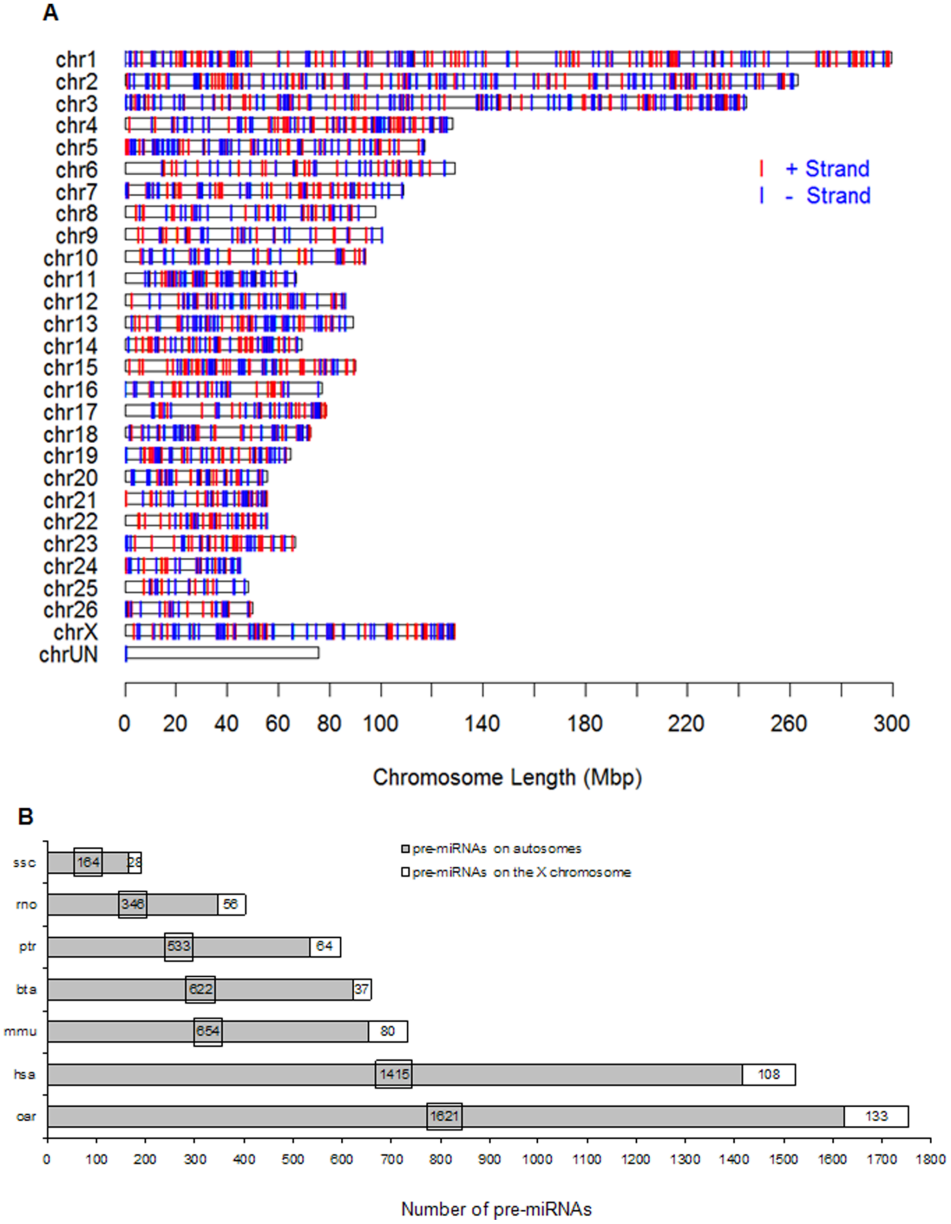
The chromosomal map of *Ovis aries* miRNAs. A. Chromosomal locations of *Ovis aries* miRNAs: miRNAs on “+ strand” (red line) and on “− strand” (blue line) across 27 pairs of chromosomes and chrUN. B. Histogram of pre-miRNAs on the X chromosome (white bar graph) and autosomes (gray bar graph) sequenced in *Ovis aries* and published in other six well-studies mammals. The chromosomal information of pre-miRNAs in these six mammals was from Sanger miRBase v18.0.

The sheep whole genome sequencing project is ongoing and the size has been estimated at 2.97 Gb. The sheep genome database (February 2010) released the base sequences of 26 pairs of autosomes and the X chromosome. After we mapped the 2,319 *Ovis aries* pre-miRNAs found in this study to all of the sheep chromosomes (see Figure 3A), we found that 1,754 pre-miRNAs were located on chromosomes and 1,879 unique miRNAs that accounted for 81.03% of the expression were mapped to 2,436 genome locations.

The number of miRNA loci on each chromosome was unequal; oar-miR-493-3p was only detected at one genome location on chromosome 18, whereas PC-3p-1818407_1 had the highest number of genome locations (23) and occurred on multiple chromosomes. An analysis of the density distribution of miRNA loci on 27 pairs of *Ovis aries* chromosomes (number of miRNA loci per Mbp of individual chromosome) indicated that the density distribution ranged from 0.46 to 2.40 miRNA loci per Mbp. In all, 2,436 miRNA loci were mapped to the *Ovis aries* genome (1,201.95 Mbp in NCBI), producing an average density of 2.03 miRNA loci per Mbp. Chromosome 6 (129.07 Mbp, possessed 60 miRNA loci) had the lowest density distribution at 0.46 miRNA loci per Mbp. Chromosome 18 (72.49 Mbp with 174 miRNA loci) had the highest density distribution at 2.40 miRNA loci per Mbp. The longest chromosome l (299.84 Mbp), and the shortest chromosome 24 (45.32 Mbp), had 212 and 64 miRNA loci, respectively, producing respective densities of 0.71 and 1.41 miRNA loci per Mbp. The number of miRNA loci on a chromosome’s positive- and negative-strands was approximately equal, at 1,327 and 1,109, respectively.

### X-linked miRNAs

Most mammals apply the XX/XY sex-determination system and no miRNAs are present on the Y chromosome. The *Ovis aries* X chromosome is 129.14 Mbp in size, ranking 4^th^ largest among all of the chromosomes. In this study, 133 pre-miRNAs were located on the X chromosome, accounting for 7.58% ( = 133/1,754) of the genome mapped pre-miRNAs (see Figure 3B). And 141 unique miRNAs were located on the X chromosome, representing 150 genome loci that accounted for 6.16% of the total miRNA loci. The density distribution of miRNA loci on the X chromosome reached 1.16 miRNA loci per Mbp, which ranked 4^th^ in the density distribution of miRNA loci among all of the chromosomes. The proportions of pre-miRNAs on the X chromosome within the total genomes of six other mammals (in Sanger miRBase v18.0) are the following: primates: human (hsa: *Homo sapiens*) 7.09%, chimpanzee (ptr: *Pan troglodytes*) 10.72%; model animals: pig (ssc: *Sus scrofa*) 14.58%, mouse (mmu: *Mus musculus*) 10.90%, rat (rno: *Rattus norvegicus*) 13.93%. However, in the cow (bta: *Bos taurus*), the proportion is only 5.61%. The distribution of miRNAs among chromosomes showed higher densities of miRNAs on X chromosomes compared to the average densities on autosomes in eight mammalian species [42], which was consistent with prior studies that demonstrated their resistance to meiotic sex chromosome inactivation [43].

### Prediction and Analysis of *Ovis aries* miRNA Target Genes

8737 *Ovis aries* miRNA target genes were predicted (see Table S22), according to the series of rules which were described in “Materials and methods”. Seen from the predicted results, one *Ovis aries* miRNA could regulate one or more target genes, i.e., bta-miR-214 predicted 319 *Ovis aries* target genes; and one *Ovis aries* target gene could be regulated by one or more miRNAs, i.e., EF462423.1 had 36 target sites of miRNAs.

### Quantitative Real-time PCR Validation of *Ovis aries* miRNAs Expression

To validate the Solexa sequencing results, 25 miRNAs (significant difference (| log_2_ | ≥0.5) and high level (counts ≥50) in the expression between Texel and Ujumqin sheep, and equal distribution in the seven types of obtained *Ovis aries* miRNAs) were selected for further analysis. These 25 *Ovis aries* miRNAs were conducted stem-loop Quantitative real-time PCR assays from the same RNA preparations used for the Solexa sequencing. All of these 25 miRNAs could be identified in Texel and Ujumqin sheep. As shown in Table 3, eight out of ten miRNAs showed similar expression patterns as those revealed by our Solexa sequencing analysis. For unknown reasons, the expression levels of bta-miR-451 and PC-3p-14023_92 were inconsistent with the Solexa sequencing results. The results confirmed that these 25 miRNAs exist in *Ovis aries*.

**Table 3:** Quantitative real-time PCR results of 10 miRNAs in Texel and Ujumqin sheep.

No.	miRNA Name	Texel NominalCT Mean	Texel NominalCT StDev	Ujumqin Nominal CT Mean	Ujumqin Nominal CT StDev	ΔΔCT	Fold Change	Up/Down
1	bta-miR-1	13.97	0.09	14.42	0.26	−0.45	1.37	Down
2	bta-miR-206_2ss16TC22GA	14.47	0.12	15.51	0.06	−1.04	2.06	Down
3	bta-miR-26a_2ss11CT21CT	16.94	0.07	17.00	0.04	−0.05	1.04	Down
4	bta-miR-378_R+1	15.75	0.07	26.94	0.10	−11.19	2,331.48	Down
5	bta-miR-451	18.14	0.16	17.91	0.19	0.23	1.18	Up
6	bta-miR-214	16.42	0.14	17.49	0.06	−1.07	2.10	Down
7	PC-3p-14023_92	25.98	0.12	25.95	0.18	0.03	1.02	Up
8	PC-5p-4553_341	23.18	0.03	23.49	0.05	−0.31	1.24	Down
9	mmu-miR-101b_R+1	24.23	0.13	24.86	0.26	−0.64	1.56	Down
10	PC-3p-36398_30	30.27	0.39	31.10	0.12	−0.83	1.78	Down

Note: Nominal CT Mean: mean value of “Nominal CT” of all members within a corresponding group; Nominal CT StDev: standard deviation value of “Nominal CT” of all members within a corresponding group; ΔΔCT = (Texel Nominal CT Mean) - (Ujumqin Nominal CT Mean); Fold Change = 2∧ABS(ΔΔCT), where ABS(ΔΔCT) is the absolute value of ΔΔCT.

## Discussion

### High Coverage of Sequencing Results

With the application of high-throughput sequencing technology, miRNA arrays can be used to identify known miRNAs, and yet they cannot detect unknown miRNAs. Deep sequencing, which takes advantage of both clone sequencing and bioinformatic prediction, is the primary method for detecting miRNAs. Deep sequencing can both detect low expression miRNAs and predict unannotated miRNAs. The Solexa Genome Analyzer, which employs Sequencing by Synthesis (SBS), is the typical second-generation sequencing instrument.

MiRNA sequencing of two small RNA libraries was performed using a Solexa Genome Analyzer. The large number of sequenced sequences ensured not only the high quality of sequence calibration, but also the comprehensive coverage of miRNA expression. The two sequence libraries included 35,700,772 raw reads that represented 34,964,457 mappable reads (15–32 nt in length; see Figure S2). Yao et al. obtained a total of 1,147,787 high-quality reads. The length distribution peaked at 22 and 23 nt, which is consistent with the results commonly expected for miRNAs [16]. The length variation was mainly affected by enzyme modifications, including RNA editing [17], 3′ editing [18], and exonuclease activity [19]–[20]; 64.07% of the mappable sequences, which accounted for 0.25% of the variation in the sequences, were identified as miRNAs or miRNA candidates (see Table S5).

### Ovis Aries miRNAome

MiRNAs are posttranscriptional regulators of gene expression; they are new targets for revealing the molecular mechanisms that form traits. Combining high-throughput sequencing and bioinformatics, this study examined 2,914 mature miRNAs representing 2,319 unique miRNAs. Among them, 1,103 unique miRNAs were conserved within Mammalia and 1,216 unique miRNAs were *Ovis aries-*specific. The count distribution of sheep miRNAs was very uneven; the first 21 miRNAs accounted for 85.06% of the total average counts (see Figure 1C). Muscle-specific miR-l, miR-133, and miR-206 all showed high expression (40,757≤ counts ≤2,699,554) in this study. Highly expressed miRNAs tended to be more stable, whereas low expression miRNAs were easily influenced by development stages. This phenomenon might relate to changes in cell regulation.

MiRNA genes are first transcribed to primary transcripts (pri-miRNAs) [21]–[25] that are further incised to about 70–90 nt hairpin precursors (pre-miRNAs). Then pre-miRNAs are processed into mature single-stranded miRNAs. Both the 5′ and 3′ ends of oar-mir-329a and oar-mir-329b matched miRNAs in miRBase v17.0, but only their 3′ ends (miR-3p) were detected in gp1a. This result agrees with previous reports in which at least 40% of miRNA sequences that were deposited in miRBase (v8.2) did not represent the predominantly cloned sequences, either because of differences in 3′ or 5′ end processing, or because of miRNA/miRNA* strand selection [26].

This study also found that the let-7 family of miRNAs possessed the same seed sequence (5′-GAGGTA-3′) reported in previous studies. Requiring conserved Watson-Crick pairing to the 5′ region of the miRNA centered on nucleotides 2–7, called the miRNA “seed”, markedly reduced the occurrence of false-positive predictions [27]–[30]. The 5′ region was the most conserved portion of metazoan miRNAs and the 5′ region of certain *Drosophila* miRNAs matched perfectly with 3′ UTR elements mediating mRNA decay and translational repression [31]. Subsequent experiments revealed that miRNA-like regulation was most sensitive to nucleotide substitutions that disrupted seed pairing [32]–[34].

Research on the miRNAome is the first step to understanding the expression, regulation, and function of miRNAs. Here, we produced a detailed overview of the *Ovis aries* miRNAome for the first time. Few papers have reported the identification of sheep miRNAs to date. These papers used bioinformatics, routine techniques, or deep sequencing based on partial tissues, and discovered 181 *Ovis aries* miRNAs [35]–[38]. Among them, 100 miRNAs (55.25%) were identical to our sequenced miRNAs and only seven miRNAs (3.87%) were not detected in our study. The remaining miRNAs had small differences from our sequenced miRNAs (see Table S21). This overlap in detected miRNAs among studies might be due to the high-expression levels of these miRNAs. However, other miRNAs showed high specificity: some miRNAs were only expressed during specific development stages, as part of certain diseases, or in specific species.

Clusters of miRNAs are also expressed as long primary transcripts. Located in a polycistron, co-expressed miRNA clusters are pivotal in coordinating the regulation of multiple processes, including embryonic development, the cell cycle, and cell differentiation [39]–[40]. By targeting components that have different roles along a signaling pathway, different members of one miRNA cluster can act as a whole to control the signal transduction process [41]. Although a large number of miRNA clusters has been discovered in animal and plant genomes, the functional consequences of this arrangement remain poorly understood.

### isomiR

Further analysis of our sequence results revealed special characters of the *Ovis aries* miRNAome (e.g., almost all of the sequenced miRNAs produced different isomiRs), illustrating that the known miRNAs are mainly present in the form of isomiRs. In this study, for a given kind of miRNA, the corresponding number of isomiRs ranged from one to hundreds, and no direct relationship was observed between the expression of a miRNA and the kinds of isomiRs, which is similar to the findings of other deep sequencing studies. Kuchenbaue et al. reported that the number of isomiRs showed only a moderate correlation with the absolute expression levels of each miRNA (R^2^ = 0.40, Pearson’s correlation coefficient), suggesting that the number of observed isomiRs was not directly related to the abundance of a miRNA. In some cases, the counts of isoforms were higher than the counts of corresponding known miRNAs in miRBase v17.0. We suggest that these most frequent isoforms should be used to refine miRBase annotations of *Ovis aries* miRNAs.

Every isomiR family has a feature and spatiotemporal specificity in certain physiological processes. More and more studies have revealed that isomiRs function in animals and may be marks of specific biogenesis processes and/or functions. This study has offered the most complete and accurate list of ovine isomiRs to date. The comprehensiveness and effectiveness of this information will set the stage for robust research into the complex functions of these regulatory molecules, which is necessary to decode the *Ovis aries* miRNAome in detail. The presence and various functions of isomiRs still require further research.

The English in this document has been checked by at least two professional editors, both native speakers of English. For a certificate, please see: http://www.textcheck.com/certificate/wi5a3Z.

## Supporting Information

Figure S1 **Data analysis flowchart.** ^1^ *Ovis aries*. ^2^Mammalia.
(TIF)

Figure S2 **Pie plot of data filtering.**
(TIF)

Figure S3 **Pie plot of database mapping.**
(TIF)

Figure S4 **Alignment of the let-7 family of miRNAs sequenced in this study and the corresponding homologous let-7 family of miRNAs recorded in miRBase v17.0.** A. Alignments of the seven kinds of sequenced let-7-5p miRNAs and the corresponding homologous let-7-5p miRNAs. “#reads (all)” was the number of all reads at 5′ end in a miRNA cluster. These let-7-5p miRNAs possessed the same seed sequence (the 2nd to 7th bases at 5′ end, 5′-GAGGTA-3′). B. Alignments of the nine kinds of sequenced let-7-3p miRNAs and the corresponding homologous let-7-3p miRNAs. “#reads (all)” was the number of all reads at 3′ end in a miRNA cluster. bta: *Bos taurus*, ssc: *Sus scrofa*, mmu: *Mus musculus*, rno: *Rattus norvegicus*, ptr: *Pan troglodytes*, hsa: *Homo sapiens*, mml: *Macaca mulatta*, cfa: *Canis familiaris*, oan: *Ornithorhynchus anatinus*, mdo: *Monodelphis domestica*, eca: *Equus caballus*, ppy: *Pongo pygmaeus*
(TIF)

Table S1 **Sample information of this study.**
(XLS)

Table S2 **Differentially and high expressed** ***Ovis aries*** **miRNAs between Texel and Ujumqin sheep.**
(XLS)

Table S3 **Primers in this study for Quantitative real-time PCR.**
(XLS)

Table S4 **Counts and types of high count (counts ≥4,718), middle count (10≤ counts <4,718) and low count (0≤ counts <10) miRNAs.**
(XLS)

Table S5 **A summary of standard data analysis results.**
(XLS)

Table S6 **Profile of known miRNAs (Group 1a) of specific species (** ***Ovis aries*** **).**
(XLS)

Table S7 **Alignment of isomiRs for known miRNAs (Group 1a) of specific species (** ***Ovis aries*** **).**
(XLS)

Table S8 **Profile of known miRNAs (Group 1b) of selected species (Mammalia) that were novel to specific species (** ***Ovis aries*** **).**
(XLS)

Table S9 **Alignment of isomiRs for known miRNAs (Group 1b) of selected species (Mammalia) that were novel to specific species (** ***Ovis aries*** **).**
(XLS)

Table S10 **Profile of predicted miRNAs (Group 2a) that could be mapped to known pre-miRNAs of selected species (Mammalia) and species specific (** ***Ovis aries*** **) genome, and within hairpins.**
(XLS)

Table S11 **Alignment of isomiRs for predicted miRNAs (Group 2a) that could be mapped to known pre-miRNAs of selected species (Mammalia) and species specific (** ***Ovis aries*** **) genome, and within hairpins.**
(XLS)

Table S12 **Profile of predicted miRNAs (Group 2b) that could be mapped to known pre-miRNAs of selected species (Mammalia) and species specific (** ***Ovis aries*** **) genome, but without hairpins.**
(XLS)

Table S13 **Alignment of isomiRs for predicted miRNAs (Group 2b) that could be mapped to known pre-miRNAs of selected species (Mammalia) and species specific (** ***Ovis aries*** **) genome, but without hairpins.**
(XLS)

Table S14 **Profile of predicted miRNAs (Group 3a) that could be mapped to known pre-miRNAs and miRNAs of selected species (Mammalia), but unmapped to species specific (** ***Ovis aries*** **) genome.**
(XLS)

Table S15 **Alignment of isomiRs for predicted miRNAs (Group 3a) that could be mapped to known pre-miRNAs and miRNAs of selected species (Mammalia), but unmapped to species specific (** ***Ovis aries*** **) genome.**
(XLS)

Table S16 **Profile of predicted miRNAs (Group 3b) that could be mapped to known pre-miRNAs of selected species (Mammalia) but unmapped to species specific (** ***Ovis aries*** **) genome.**
(XLS)

Table S17 **Alignment of isomiRs for predicted miRNAs (Group 3b) that could be mapped to known pre-miRNAs of selected species (Mammalia) but unmapped to species specific (** ***Ovis aries*** **) genome.**
(XLS)

Table S18 **Profile of predicted miRNAs (Group 4a) that were unmapped to known miRNAs, but mapped to species specific (** ***Ovis aries*** **) genome and within hairpins.**
(XLS)

Table S19 **Alignment of isomiRs for predicted miRNAs (Group 4a) that were unmapped to known miRNAs, but mapped to species specific (** ***Ovis aries*** **) genome and within hairpins.**
(XLS)

Table S20 **Overall** ***Ovis aries*** **unique miRNAs sequenced in this study.**
(XLS)

Table S21 **Comparison of** ***Ovis aries*** **new identified miRNAs between other literatures and our study.**
(XLS)

Table S22 **The information of predicted target genes for** ***Ovis aries*** **miRNAs.**
(XLS)
