# Supplementary figures and images for: Identification and Characterization of the miRNA Transcriptome of Ovis aries
Source: PLoS One. 2013 Mar 13;8(3):e58905. doi: 10.1371/journal.pone.0058905 (PMC3596360; doi:10.1371/journal.pone.0058905)

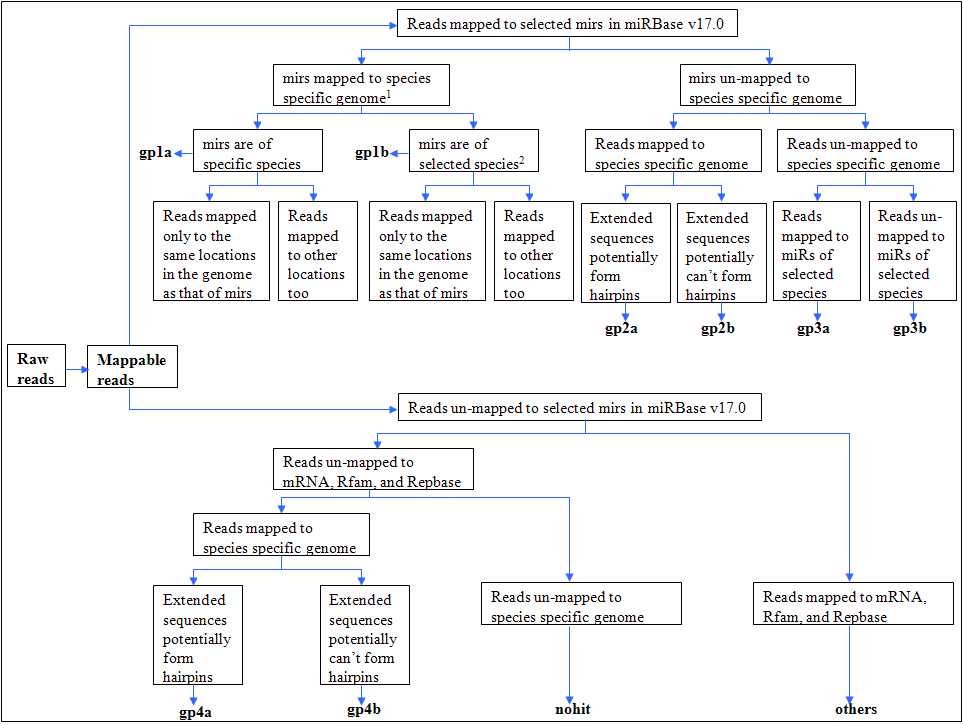

Supplement: Figure S1 — Data analysis flowchart. 1 Ovis aries. 2Mammalia. (TIF) [file pone.0058905.s001.tif]

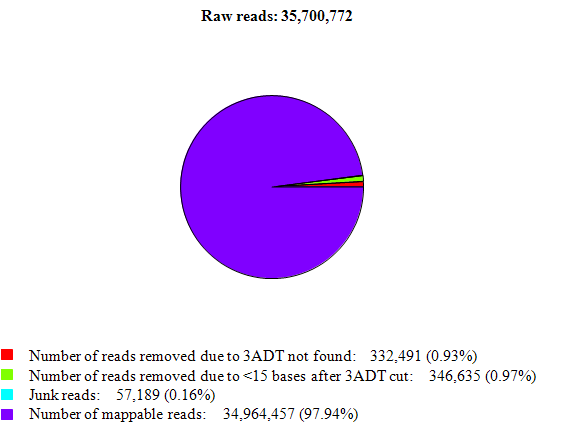

Supplement: Figure S2 — Pie plot of data filtering. (TIF) [file pone.0058905.s002.tif]

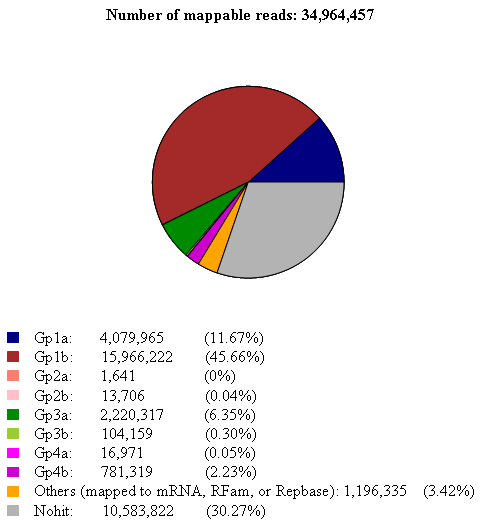

Supplement: Figure S3 — Pie plot of database mapping. (TIF) [file pone.0058905.s003.tif]

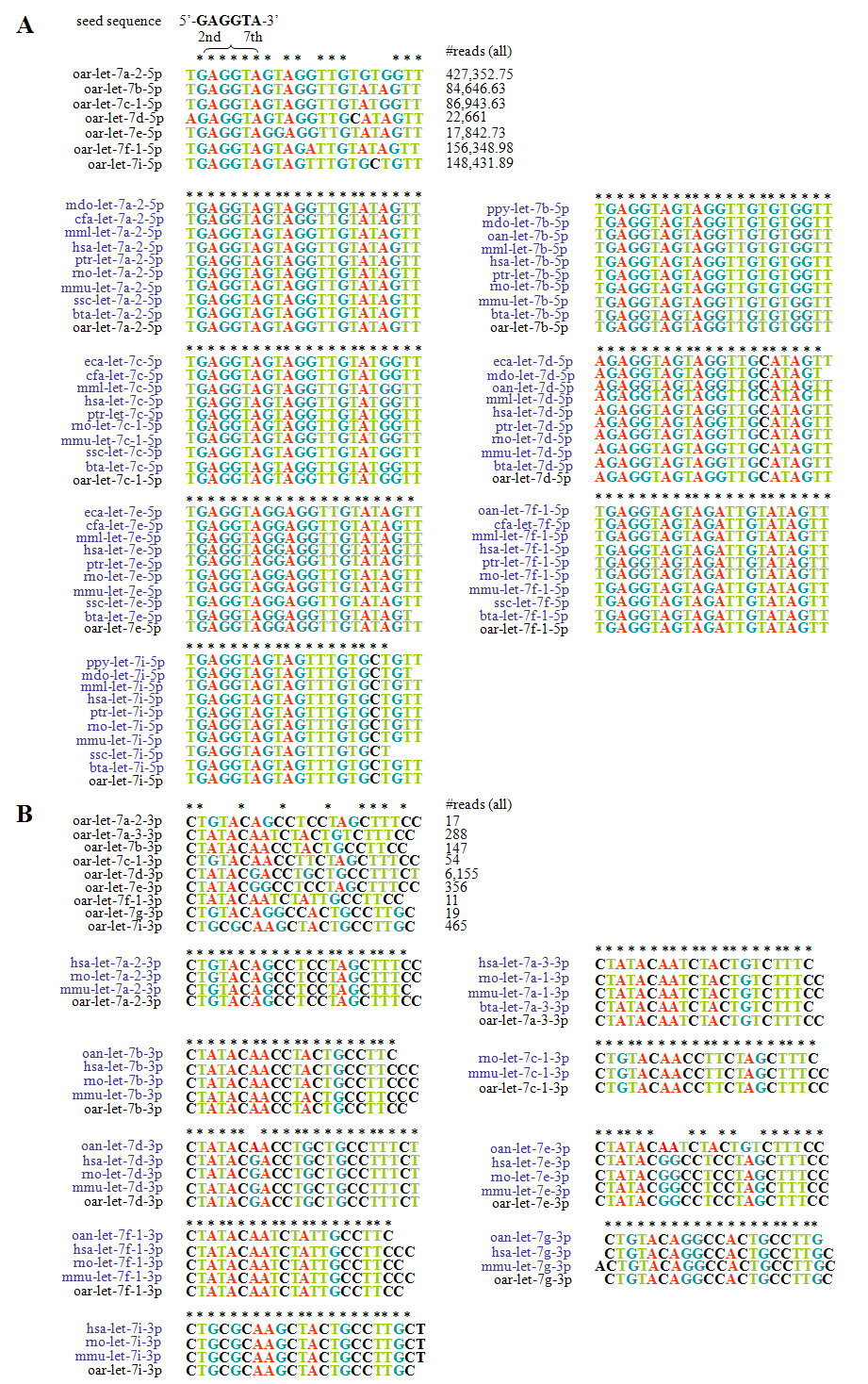

Supplement: Figure S4 — Alignment of the let-7 family of miRNAs sequenced in this study and the corresponding homologous let-7 family of miRNAs recorded in miRBase v17.0. A. Alignments of the seven kinds of sequenced let-7-5p miRNAs and the corresponding homologous let-7-5p miRNAs. “#reads (all)” was the number of all reads at 5′ end in a miRNA cluster. These let-7-5p miRNAs possessed the same seed sequence (the 2nd to 7th bases at 5′ end, 5′-GAGGTA-3′). B. Alignments of the nine kinds of sequenced let-7-3p miRNAs and the corresponding homologous let-7-3p miRNAs. “#reads (all)” was the number of all reads at 3′ end in a miRNA cluster. bta: Bos taurus, ssc: Sus scrofa, mmu: Mus musculus, rno: Rattus norvegicus, ptr: Pan troglodytes, hsa: Homo sapiens, mml: Macaca mulatta, cfa: Canis familiaris, oan: Ornithorhynchus anatinus, mdo: Monodelphis domestica, eca: Equus caballus, ppy: Pongo pygmaeus (TIF) [file pone.0058905.s004.tif]
